# The effects of perfluorooctanoic acid (PFOA) on physiological processes and oxidative damage in edible vegetables

**DOI:** 10.1515/biol-2025-1250

**Published:** 2026-01-23

**Authors:** Hakan Ozden

**Affiliations:** Department of Biology, Division of Botany, Faculty of Science, Istanbul University, Istanbul, Türkiye

**Keywords:** perfluorooctanoic acid, plant physiological processes, oxidative damage, lettuce, radish, carrot

## Abstract

Perfluorooctanoic acid (PFOA) is widely utilized perfluoroalkyl substance (PFAS) in various industrial and household applications, known for its persistence in the environment. Due to the limited toxicity data for PFOA in edible vegetable plants, this study aimed to examine its phytotoxicity on growth and physiological processes of PFOA (0, 10, 50, 100 µM) exposure for 14 days in lettuce (*Lactuca sativa*), radish (*Raphanus sativus*), and carrot (*Daucus carota*). Growth rates of lettuce and radish were altered at 10–100 µM of PFOA, whereas no significant changes were observed in carrots, and a dose-dependent reduction in chlorophyll content was observed. Malondialdehyde (MDA) levels in lettuce and radish were elevated by at least 1.70-fold at 50 and 100 µM. PFOA was found to trigger an adaptive effect in seedlings, with a reduction of at least 30.58 % in reduced glutathione (GSH) content in lettuce and radish at 100 µM. However, no alterations in MDA or GSH levels were observed in carrot, suggesting the possibility that it may be resistant to the toxic effects of PFOA. Overall, our research suggests that PFOA exhibits toxic effects on edible plants, particularly lettuce and radish, affecting their growth, development, and physiological processes while inducing oxidative damage.

## 1 Introduction

Perfluorooctanoic acid (PFOA) is a synthetic chemical classified under the broader group of per- and polyfluoroalkyl substances (PFAS). It has been extensively used in a range of industrial and consumer products, such as non-stick cookware, stain-resistant textiles, food packaging, and firefighting foams [1], 2]. PFOA is moderately soluble in water, has low volatility, and is highly persistent in both the environment and living organisms, leading to significant concerns about widespread contamination and bioaccumulation [3], 4]. PFOA can accumulate in plant tissues and potentially enter the food chain when consumed by animals or humans. The consumption of contaminated vegetables can result in human exposure to PFOA, which has been associated with various health issues, including cancer, liver damage, and developmental problems [5], 6]. The International Agency for Research on Cancer (IARC) has classified PFOA as a possible human carcinogen (Group 2B) [7]. Given these potential risks, regulatory agencies have implemented limits on PFOA levels in the environment and food products. Assessing the toxicity of PFOA in vegetables is crucial for developing effective strategies to protect public health. Recent attention has been drawn to the limited research on the toxicity of PFAS in plants. Available evidence indicates that PFOA may have adverse effects on plant growth and development, including reduced seed germination, inhibited root and shoot growth, and altered leaf morphology [8], [9], [10]. Furthermore, PFOA can disrupt nutrient uptake in plants, adversely affecting their overall nutrient status and metabolic processes.

PFOA’s toxic impact on plants is primarily linked to its ability to disrupt cellular functions. It has been demonstrated that PFOA induces oxidative stress, which leads to the production of reactive oxygen species (ROS) [9], [10], [11]. This, in turn, damages plant cell membranes. Additionally, PFOA has been found to interfere with hormonal regulation in plants [12], affecting key physiological processes such as photosynthesis and water uptake. A diverse range of agricultural products, including crops, vegetables, and fruits from PFAS-contaminated regions, have been found to contain significant amounts of PFASs [13], [14], [15], [16]. It has been documented that leafy and root vegetables tend to accumulate higher levels of PFOA in their edible parts compared to fruit and fruit vegetables [6], 17]. Research on the phytotoxicity of PFOA in plants that produce edible vegetables is essential for conducting a comprehensive risk assessment. Lettuce (*Lactuca sativa*), one of the most widely cultivated and consumed leafy vegetables, was selected as the model plant, as indicated by several studies [11], [18], [19], [20]. Additionally, radish (*Raphanus sativus*) and carrot (*Daucus carota*) were chosen to investigate the phytotoxic effects of PFOA, as these vegetables have not been extensively studied in this context. Chemical exposure to seed prior to germination may influence early developmental processes due to potential interference with critical cellular reprogramming and metabolic activation phases. Therefore, this study investigated the phytotoxic effects of PFOA on leafy and root vegetables, specifically lettuce, radish, and carrot, by exposing them to varying concentrations of PFOA either before germination or after the germination period for 14 d. In particular, the research aimed to highlight the potential impacts of PFOA on agriculture and food safety and to provide scientific data for the risk assessment of PFOA in agricultural practices and environmental systems. Understanding the effects of PFOA on plant systems, especially edible vegetables, is essential for evaluating its risks to both food safety and human health.

## 2 Materials and methods

### 2.1 Chemicals

PFOA (95 % purity) was obtained from Sigma Aldrich (Munich, Germany). A stock solution of PFOA (1,000 µM) was prepared by dissolving it in distilled water in a polypropylene container and kept at −20 °C. All reagents were purchased from Sigma-Aldrich (St. Louis, MO, USA).

### 2.2 Plant growth and exposure to PFOA

Lettuce (*L. sativa*), radish (*R. sativus*), and carrot (*D. carota*) seeds were supplied from a local nursery. Prior to germination, the seeds were carefully cleaned to remove any debris. Surface sterilization was performed using 5 % sodium hypochlorite for 5 min, followed by three to four rinses with distilled water to remove any residual sterilizing agent. The seeds were then pre-germinated for 5 d at 4 °C in petri dishes containing two layers of sterile filter paper saturated with ultrapure water. Following pre-germination, 10 seeds of each plant species per group were transferred to individual Petri dishes assigned to eight treatment groups, including control groups, and were cultivated in Ingestad nutrient solution. In Group A, pre-germinated seeds were exposed to PFOA at concentrations of 0, 10, 50, and 100 µM for 14 d in a controlled climate chamber. In Group B, pre-germinated seeds were first allowed to grow for 7 d, after which the developing seedlings were subjected to PFOA exposure (0, 10, 50, and 100 µM) for an additional 14 d in the same controlled conditions. The climate chamber was set to the following conditions: a photoperiod of 16 h of light and 8 h of darkness; a light intensity of 222,22 μmol/m^2^/s; a temperature of 25 ± 2 °C; and a relative humidity of 65 ± 2 %. Control groups were performed using distilled water instead of PFOA exposure under the same study conditions. The selected PFOA concentrations were based on prior research [5], [20], [21], [22]. Each concentration group consisted of 10 seeds in a Petri dish and was evaluated in three independent experiments, with each analysis repeated twice. To prevent microbial contamination, the Ingestad nutrient solution was refreshed every 3–4 d. At the end of the 14 d exposure period, samples from both Group A and Group B were collected to assess germination rates, growth development, and the concentrations of chlorophyll, malondialdehyde (MDA), and glutathione (GSH) in the seedlings.

### 2.3 Measurement of plant physiological parameters

The seeds used in the study were placed in Petri dishes assigned to control and treatment groups in specified numbers. At the end of the treatment period, the seed germination rate was calculated as a percentage based on the number of seeds that had germinated successfully in each Petri dish compared to the control samples. The plant growth rate was determined by measuring root elongation in both the control and treatment groups; these measurements were then compared to those of the control group in order to evaluate the relative growth response. Root lengths were measured using millimeter paper, with measurements verified using an electronic caliper for accuracy in the control and treatment groups. The fresh weights of roots were measured for the exposed and control samples following the procedure outlined by Horwitz [23].

### 2.4 Measurement of chlorophyll and carotenoid contents

The chlorophyll and carotenoid content in lettuce, radish, and carrot seedlings was determined using a spectrophotometric method by Lichtenthaler and Wellburn [24]. 500 mg of each fresh leave were prepared by cutting them into pieces of uniform size, then were homogenized using an Ultraturrax device (Janke & Kunkel, Germany) in the presence of calcium carbonate and ice-cold acetone (100 %, v/v). The resulting homogenates were centrifuged at 3,000 g for 10 min (Heraeus Labofuge 400 R, Germany), and the supernatants were collected for further analysis. Absorbance readings at 662 nm, 645 nm, and 470 nm were recorded spectrophotometrically (Jenway 6105 UV–Vis Spectrophotometer, Great Britain) to determine the concentrations of chlorophyll a, chlorophyll b, and total carotenoids, respectively. The concentrations of chlorophyll and carotenoids were calculated using the formulas provided by Lichtenthaler and Wellburn [24]. The results were expressed in mg/g fresh weight (fw).

$$\text{Chlorophyll}-a=\left(11.75\times A662-2.35\times A645\right)\times 10/g\text{fw}$$

$$\text{Chlorophyll}-b=\left(18.61\times A645-3.96\times A662\right)\times 10/g\text{fw}$$

$$\text{Total carotenoids}=\left[\left(1000\times A470-2.27\times \text{Chl}-a-81.4\times \text{Chl}-b\right)/227\right]\times 10/g\text{fw}$$

### 2.5 Measurement of oxidative stress parameters

Malondialdehyde (MDA) levels, measured as an indicator of lipid peroxidation and reflecting oxidative damage, were quantified using the thiobarbituric acid (TBA) reaction method, in which MDA reacts with TBA to form coloured complex under high-temperature and acidic conditions [25]. 500 mg of fresh leaves were extracted with 0.1 % trichloroacetic acid (TCA), and the reaction with TBA was carried out at 95 °C. Absorbance was measured at 532 and 600 nm spectrophotometrically, MDA content was calculated using the formula below, and results were expressed as nmol/g fresh weight (fw):

$$\text{MDA}\left(\text{nmol}/g\text{fw}\right)=\left[\left(A532-A600/155mM^{-1}{\text{cm}}^{-1}/\text{fw}\left(g\right)\right)\times 10^{6}\right]$$

Glutathione content was determined using the 5,5-dithiobis-(2-nitrobenzoic acid) (DTNB) reagent, according to the method described by Sedlak and Lindsay [26]. Briefly, 500 mg of fresh leaves were extracted using 5 % sulfosalicylic acid, and total glutathione levels were measured spectrophotometrically at 412 nm. Total glutathione contents were calculated using a glutathione standard curve, and the results were expressed as nmol/g fw.

### 2.6 Statistical analysis

All data were presented as mean ± standard deviation. Statistical evaluations were performed using “SPSS 21.0 for Windows” software. A “One-Way Analysis of Variance (ANOVA)” was applied, followed by Tukey’s Post Hoc test. A p-value of less than 0.05 or 0.001 was considered statistically significant.

## 3 Results and discussion

Exposure to chemicals before germination may have a more profound effect on developmental processes, as it interferes with cellular reprogramming and metabolic activation at an earlier stage than post-germination applications. Therefore, in this study, edible plants were exposed to PFOA under two different conditions – pre- and post-germination – to evaluate its effect on plant growth, physiology, and oxidative damage.

### 3.1 Effects of PFOA on plant growth conditions and plant physiology

**Figure 1:**
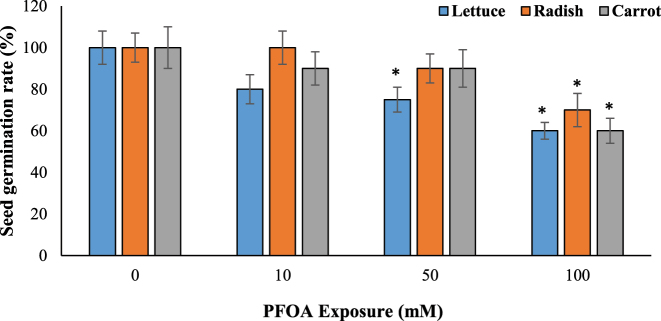
Effects of PFOA exposure (0–100 µM) on seed germination (%) in lettuce, radish and carrot. Data are presented as mean ± SD Statistically significant changes are indicated by *p < 0.05 (ANOVA pot hoc Tukey test).

Germination is a critical phase in the life cycle of plants, and any adverse effects during this stage can significantly impact crop yields. We evaluated the impact of PFOA on seed germination in Group A, where we observed that the seed germination rates of lettuce, radish, and carrot were significantly reduced (by 30–40 %, p < 0.05) at a concentration of 100 µM of PFOA compared to the control group (Figure 1). The decrease was dose-dependent, with higher concentrations causing more significant effects, as demonstrated in the following studies. The germination rate and germinability of Chinese cabbage were reduced in a dose-dependent manner by PFOS contamination (34–85 mg/g) [21]. Additionally, germination rate, root, and shoot growth of *Triticum aestivum* L. were adversely affected by >800 mg/kg PFOA [10].

**Figure 2:**
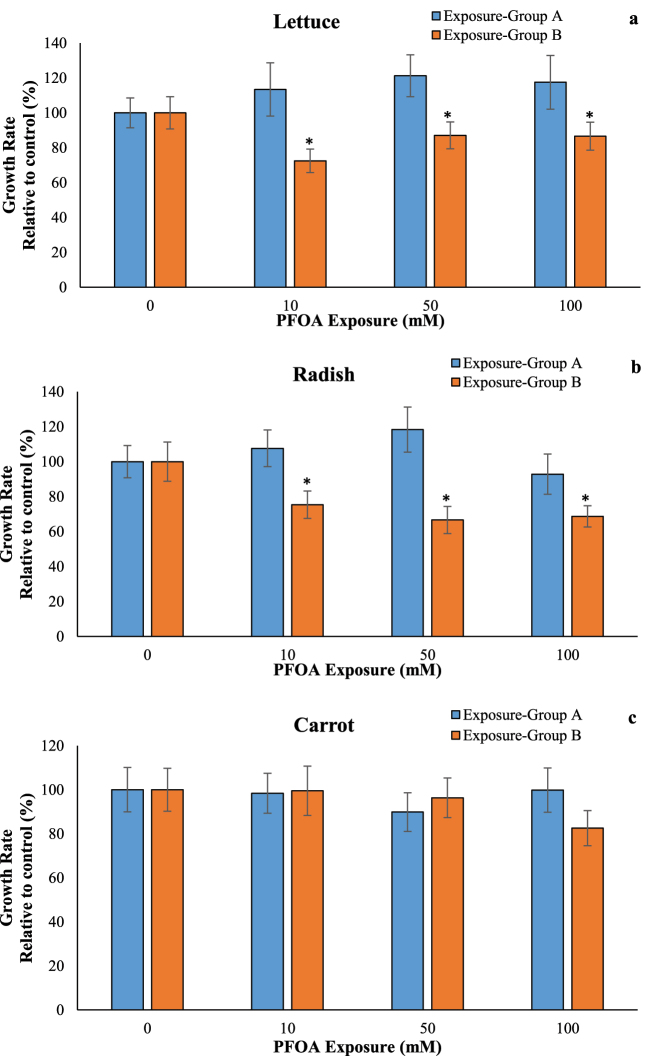
Effects of PFOA exposure (0–100 µM) on growth rate in lettuce (a), radish (b) and carrot (c) in the exposure Group A and Group B. Data are presented as mean ± SD statistically significant changes are indicated by *p < 0.05 (ANOVA pot hoc Tukey test). Exposure Group A: PFOA exposure was initiated prior to germination and continued for a duration of 14 d, Group B: PFOA exposure for 14 d following a 7 d germination period.

There are non-significant changes in the growth rate of lettuce, radish, and carrot at the exposure to PFOA for 14 d in Group A (Figure 2). Exposure to 10, 50, and 100 µM of PFOA after germination (Group B) led to a significant decrease in the growth rate of lettuce and radish, with reductions of at least 12.94 % (p < 0.05) and 24.64 % (p < 0.05), respectively, while no changes were observed in carrot. Chen et al. [5] demonstrated that exposure to 5 mg/L of PFOA for 21 d resulted in significant growth inhibition in *Arabidopsis thaliana* and *Nicotiana benthamiana*. In another study, the growth rate of *A.thaliana* seedlings was significantly inhibited at 50 µM of PFOA, with the inhibitory effects becoming more pronounced at higher PFOA concentrations [22]. Additionally, exposure to 20, 50, and 100 µM of PFOA resulted in reductions of approximately 20 %, 43 %, and 61 %, respectively, in shoot growth of *A. thaliana* [22]. Exposure to 18 µM PFOA-F had no effect on plant growth, whereas concentrations of 181–1,811 µM PFOA-F reduced both root and shoot growth in *A. thaliana* [9].

**Figure 3:**
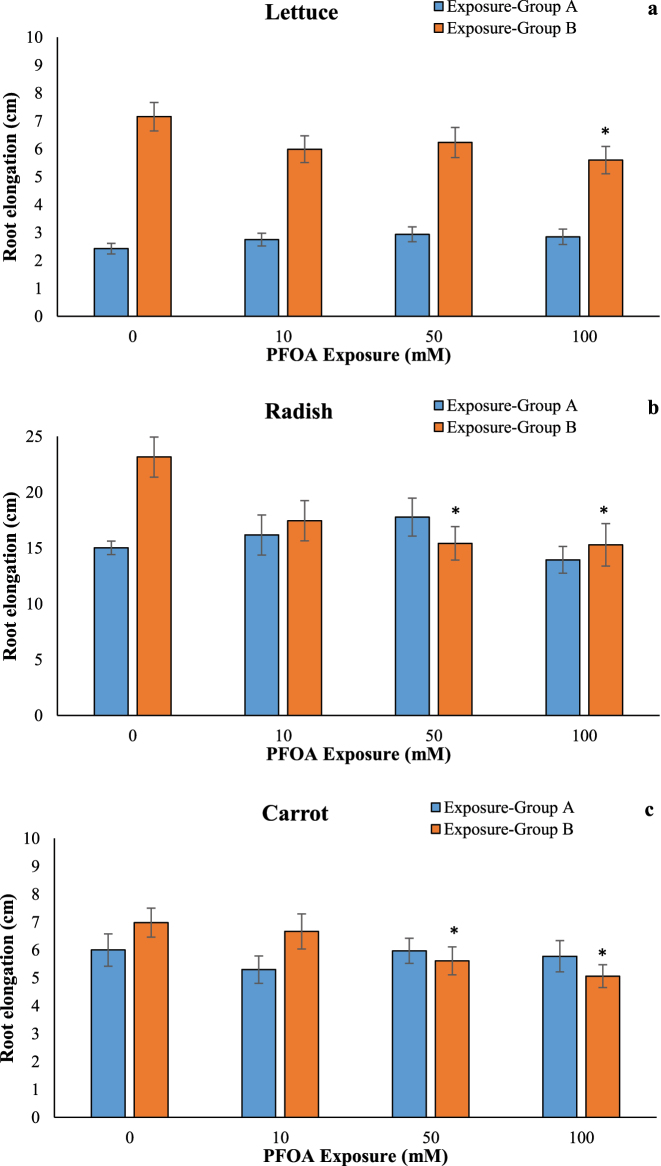
Effects of PFOA exposure (0–100 µM) on root elongation in lettuce (a), radish (b) and carrot (c) in the exposure Group A and Group B. Data are presented as mean ± SD statistically significant changes are indicated by *p < 0.05 (ANOVA pot hoc Tukey test). Exposure Group A: PFOA exposure was initiated prior to germination and continued for a duration of 14 d, Exposure Group B: PFOA exposure for 14 d following a 7 d germination period.

Root elongation of lettuce, radish, and carrot was significantly reduced by at least 17.88 % at 100 µM of PFOA in the exposure Group B (Figure 3), while no changes were observed in Group A. Exposure to 20–200 µM of PFOA resulted in a decrease in primary root length by approximately 5–22 % compared to the control in *A. thaliana* [22]. Zhou et al. [10] reported that root biomass and length were significantly inhibited in wheat (*T. aestivum* L.) seedlings under exposure to 800 mg/kg of PFOA [10]. Exposure to 181–1,811 µM of PFOA-F inhibited both root and shoot growth in *A. thaliana* [9]. Li (2009) found that 62.5–2000 mg/L of PFOA significantly affected the root elongation of lettuce (*L. sativa*), pakchoi (*Brassica rapa subsp. chinensis*), and cucumber (*Cucumis sativus*) [8]. As indicated by these studies, PFOA exposure may inhibit root growth, leading to stunted root systems and shorter shoot lengths in seedlings exposed to PFOA. This could potentially affect the plant’s ability to uptake water and nutrients. It is noteworthy that Felizeter et al. observed that exposure to PFOA concentrations of 0.01–10 μg/L had no remarkable impact on the growth of lettuce (*L. sativa*) [18]. Similarly, Li and Li reported that exposure to 5 μg/L and 50 μg/L of PFOA for 10 d did not affect the root lengths of lettuce [11].

**Figure 4:**
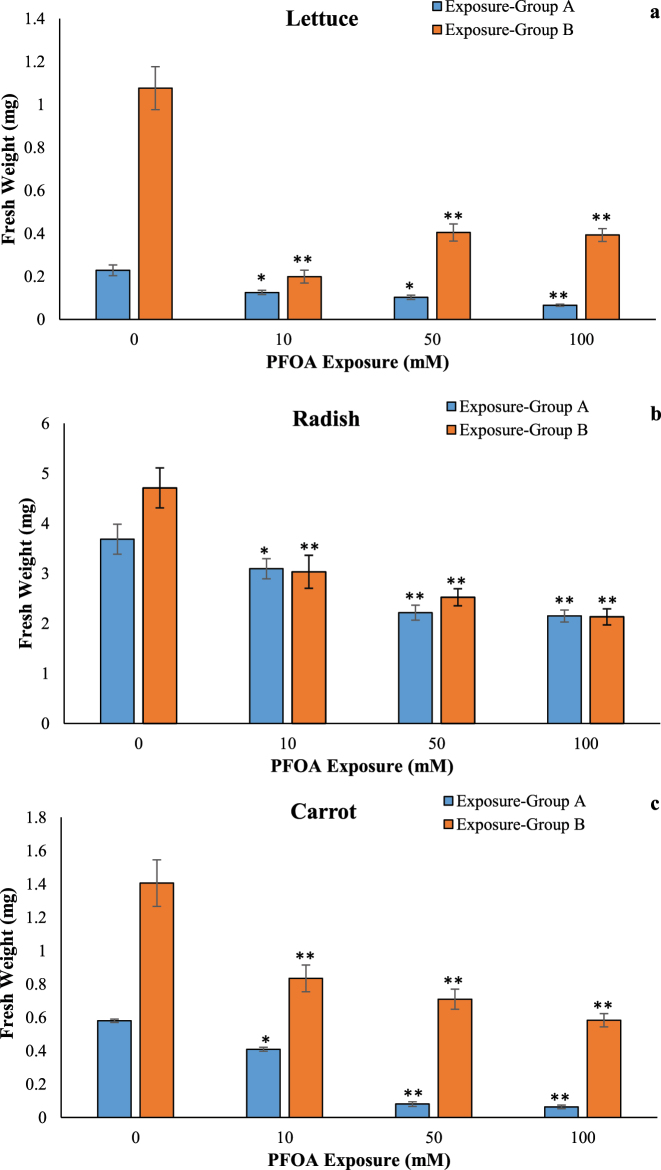
Effects of PFOA exposure (0–100 µM) on fresh weight in lettuce (a), radish (b) and carrot (c) in the exposure Group A and Group B. Data are presented as mean ± SD statistically significant changes are indicated by *p<0.05, **p < 0.001 (ANOVA pot hoc Tukey test). Exposure Group A: PFOA exposure was initiated prior to germination and continued for a duration of 14 d, Exposure Group B: PFOA exposure for 14 d following a 7 d germination period.

The fresh weight of seedlings of lettuce, radish, and carrot decreased by at least 45.05 %, 16.03 %, and 29.48 %, respectively, at 10–100 µM of PFOA in the exposure Group A (Figure 4) according to the control group. In Group B exposure to 10–100 µM of PFOA caused a decrease by at least 62.41 %, 35.62 %, and 40.65 %, respectively, in the fresh weight of seedlings of lettuce, radish and carrot according to the control group (Figure 4). It is commonly observed that the overall fresh weight of seedlings, including the root system, is reduced in plants exposed to PFOA. This decrease in fresh weight may impact the plant’s vigor and productivity. Our observations were consistent with previous findings regarding the effects of PFOA on plant growth [22]. Especially, as it can be seen, the lettuce is more vulnerable to the phytotoxic effects of PFOA than radish and carrot.

### 3.2 Effects of PFOA on chlorophyll contents

**Figure 5:**
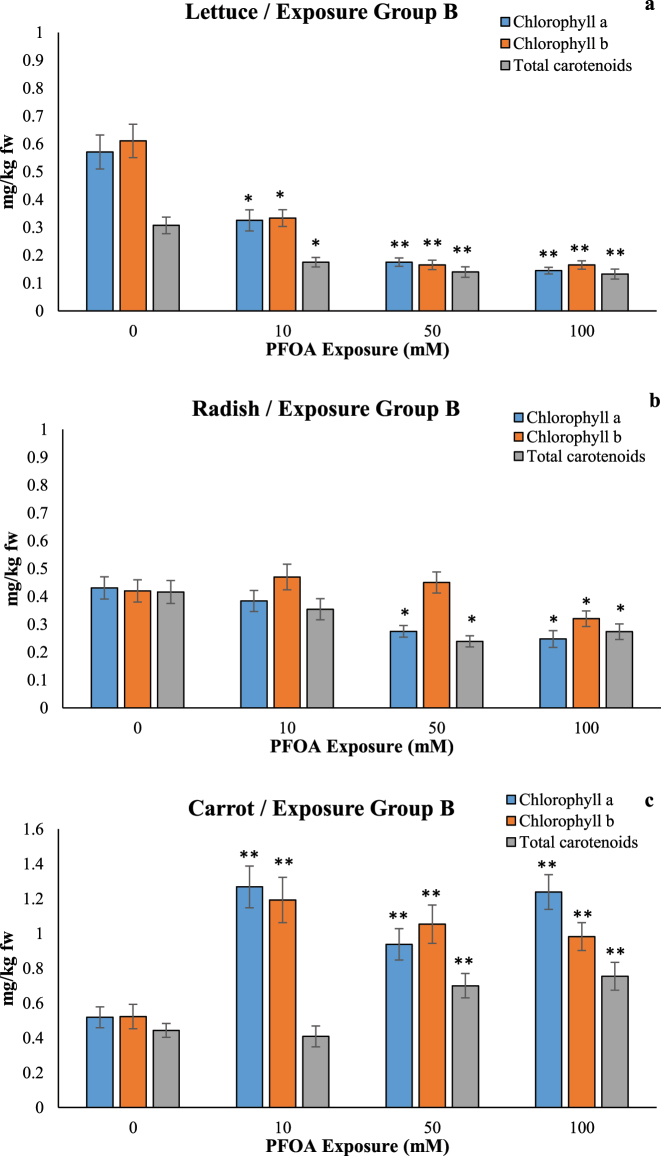
Effects of PFOA exposure (0–100 µM) on chlorophyll a, chlorophyll b and carotenoids in lettuce (a), radish (b) and carrot (c) in the exposure Group A and in lettuce (e), radish (f) and carrot (g) in the exposure Group B. Data are presented as mean ± SD statistically significant changes are indicated by *p < 0.05, **p < 0.001 (ANOVA pot hoc Tukey test). Exposure Group B: PFOA exposure for 14 d following a 7 d germination period.

The content of chlorophyll a, chlorophyll b, and carotenoids decreased in a dose-dependent manner, especially at 100 µM PFOA in lettuce seedlings, with a decrease of 44.44 %, 40 %, and 47.03 % and in radish seedlings, with a decrease of 43.58 %, 33.33 %, and 32.43, respectively, observed in exposure group A, while significant decreases of at least 74.66 %, 72.99 %, and 56.88, respectively, were also observed at 100 µM PFOA in exposure group B. (Figure 5). However, in carrot seedlings, the chlorophyll a, chlorophyll b, and carotenoid content at 100 µM of PFOA increased by 1.56-fold, 1.72-fold, and 1.73-fold, respectively, in exposure group A and 1.70-fold, 2.39-fold, and 1.88-fold, respectively, in exposure group B (Figure 5). Li et al. demonstrated that following exposure to 500 ng/L PFOA, 500 ng/L, and 5,000 ng/L PFOS in lettuce leaves, the total photosynthetic pigments (chlorophyll a, b, and carotenoids) increased by 15.1 %, 28.3 %, and 16.3 %, respectively [20]. Exposure to PFOA can negatively affect photosynthetic efficiency, influencing chlorophyll content and the overall photosynthetic capacity of the plant. This reduction in photosynthetic pigments can inhibit plant growth and development. Consistently, 20 μg/kg of PFOA exposure for 60 days reduced the content of chlorophyll a and b and carotenoids in radish plant [27].

According to Qu et al., exposure to 10 mg/L PFOS resulted in damage to chlorophyll accumulation in wheat (*T. aestivum* L.) [28]. A dose-dependent decrease in chlorophyll levels was significantly observed after 2 weeks of growth in *A. thaliana* exposed to 20, 50, 100, and 200 µM PFOA [22]. PFOA exposure decreased total chlorophyll content by 39.4 % at 10 mg/L, while chlorophyll a and chlorophyll b were unaffected at 1 mg/mL in the leaves of *Acorus calamus* seedlings [29]. Chlorophyll levels in wheat were inhibited by 21.92 % following treatment with PFAS [30]. Another study found that chlorophyll levels decreased by 24.8–37.8 % and 28.9–38.3 % in *A. calamus* and *Phragmites communis*, respectively, after 12 and 24 d of exposure to 50 mg/L PFOS [31]. Previous studies suggest that the inhibition of chlorophyll synthesis by PFASs may be related to chloroplast electron transfer during photosynthesis, although the specific mechanisms involved need further investigation [30]. In addition, chlorophyll content did not increase or decrease linearly with rising PFOS concentrations [31], which may be attributed to the fluctuations in chlorophyll being a result of an imbalance between its synthesis and degradation processes [32].

### 3.3 Effects of PFOA on oxidative damage

**Figure 6:**
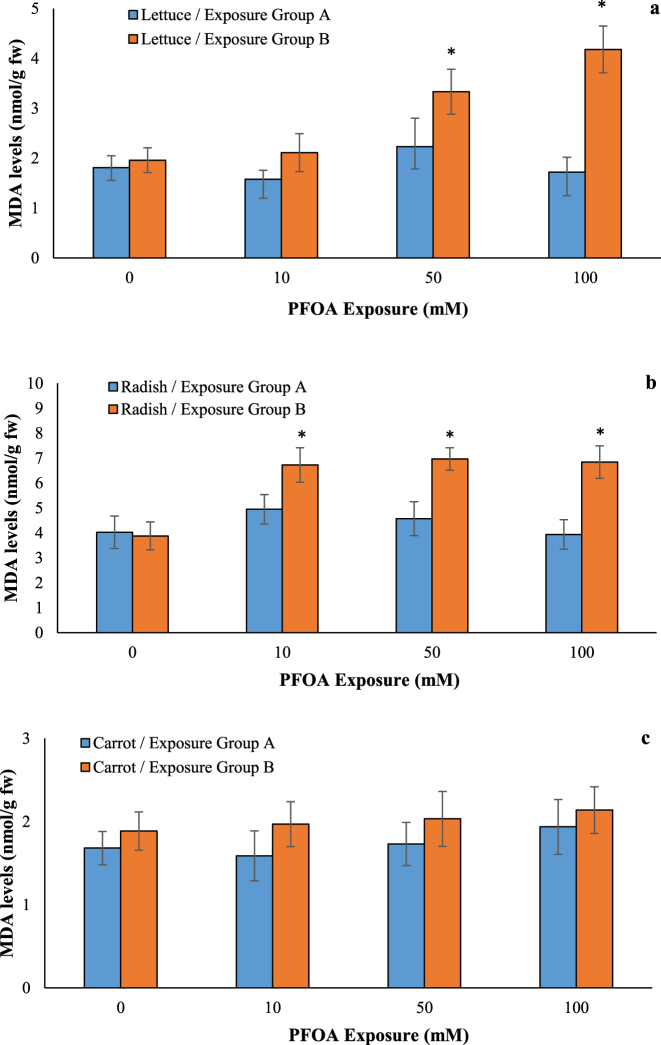
Effects of PFOA exposure (0–100 µM) on MDA levels in lettuce (a), radish (b) and carrot (c) in the exposure Group A and Group B. Data are presented as mean ± SD statistically significant changes are indicated by *p < 0.05 (ANOVA pot hoc Tukey test). Exposure Group A: PFOA exposure was initiated prior to germination and continued for a duration of 14 d, Exposure Group B: PFOA exposure for 14 d following a 7 d germination period.

**Figure 7:**
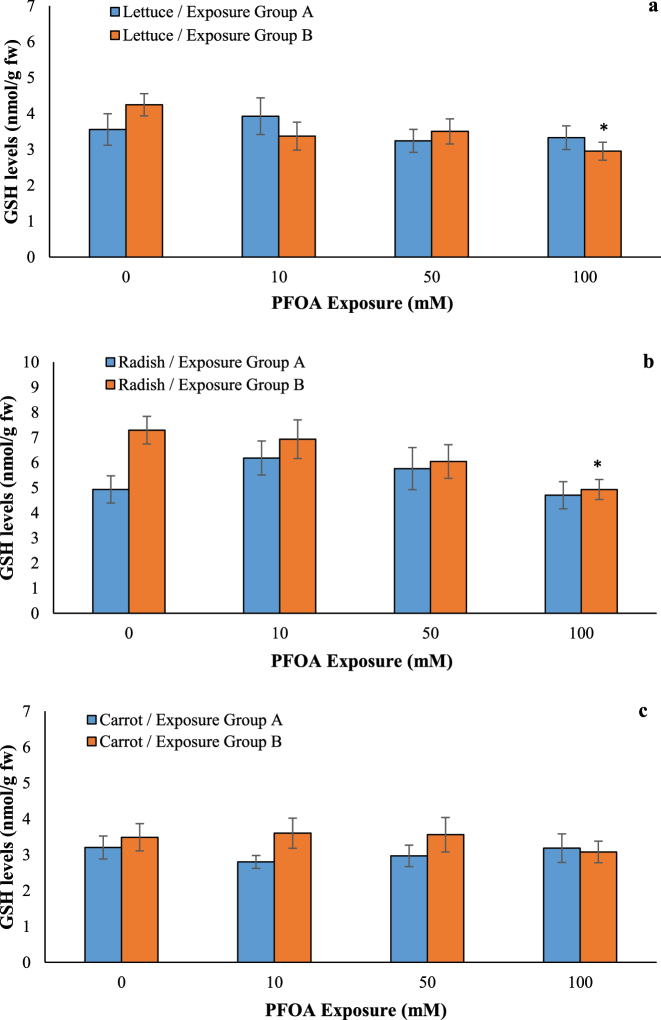
Effects of PFOA exposure (0–100 µM) on GSH levels in lettuce (a), radish (b) and carrot (c) in the exposure Group A and Group B. Data are presented as mean ± SD statistically significant changes are indicated by *p < 0.05 (ANOVA pot hoc Tukey test). Exposure Group A: PFOA exposure was initiated prior to germination and continued for a duration of 14 d, Exposure Group B: PFOA exposure for 14 d following a 7 d germination period.

Free radical overproduction can initiate lipid peroxidation, which in turn leads to oxidative damage [33]. Non-enzymatic antioxidants like GSH play a crucial role in scavenging ROS [34]. MDA levels increased by at least 1.70-fold at 50 and 100 µM of PFOA in lettuce seedlings and by at least 1.73-fold at 10, 50, and 100 µM of PFOA in radish seedlings in exposure Group B (Figure 6), while no significant change was observed in exposure Group A. Exposure to 100 µM PFOA reduced GSH content by 30.58 % in lettuce and 32.37 % in radish (Figure 7) in Group B, whereas no change was observed in the exposure Group A. By contrast, no change in MDA and GSH levels was observed in PFOA-exposed carrot seedlings in both exposure groups, suggesting a possible adaptive effect in carrots, but not lettuce and radish. Exposure to PFOA leads to the generation of ROS, resulting in oxidative stress that damages biological components [11]. Consistent with our results, Du et al. reported that PFOA (20 μg/kg) caused significant oxidative stress in radish leaves, as evidenced by elevated MDA content [27]. Li et al. [35] indicated that MDA contents were significantly increased 50.0–57.8 % in leaves of lettuce after 5–50 μg/L PFOA exposure, indicating the occurrence of lipid peroxidation. In addition, 50 μg/L of PFOA resulted in a 54.2 % increase in GSH levels in lettuce leaves, indicating that GSH plays a critical role in eliminating ROS after PFOA exposure [35]. Lipid peroxidation was observed in *A. calamus* leaves under high concentrations of PFOA stress (10 mg/L), with MDA accumulation increasing by 1.20–1.22 times compared to the control [29]. Similarly, PFOA at 725 μM significantly increased MDA levels by 45 % in the shoots of *A. thaliana*, but no change was observed in the roots [9]. Despite the non-significant ROS overproduction, GSH levels increased by 88.9 % in lettuce after 10 d of exposure to 50 μg/L PFOA [11]. In contrast, no changes in GSH levels were observed in the roots of *A. thaliana* exposed to 20–100 μM PFOA [22]. These findings suggest that the accumulation of PFASs in plants can induce membrane lipid peroxidation via MDA, leading to damage to cellular structures and macromolecules, ultimately causing phytotoxicity.

## 4 Conclusions

In the present study, the stress responses to PFOA exposure in lettuce, radish, and carrot seedlings were evaluated by investigating their physiological processes and oxidative damage after pre-germination and post-germination exposure conditions. Our findings suggest that PFOA inhibits plant growth and pigment content while inducing oxidative damage and alterations in physiological parameters in lettuce and radish, with minimal effects observed in carrots. However, exposure to PFOA before and after germination did not appear to make a difference in the toxic effects of PFOA. This outcome suggests that the phytotoxic effects of PFOA are primarily concentration-dependent rather than timing-dependent, indicating a persistent mode of action regardless of the plant’s developmental stage. These results provide valuable insights for assessing the ecological risks of PFASs and the potential impact on the nutritional value of agricultural products, contributing to future research in this area. Efforts to reduce the use of PFAS and develop environmentally friendly alternatives remain critical to protecting both ecosystems and human health.
